# Complete Genome Sequence of a Novel Mutant Strain of Vibrio parahaemolyticus from Pacific White Shrimp (Penaeus vannamei)

**DOI:** 10.1128/genomeA.00497-18

**Published:** 2018-06-14

**Authors:** Siddhartha Kanrar, Arun K. Dhar

**Affiliations:** a Aquaculture Pathology Laboratory, School of Animal and Comparative Biomedical Sciences, The University of Arizona, Tucson, Arizona, USA

## Abstract

The acute hepatopancreatic necrosis disease (AHPND) of Penaeus vannamei shrimp is caused by Vibrio parahaemolyticus carrying toxin genes, *pirA* and *pirB*. We report the complete genome sequence of the novel V. parahaemolyticus strain R14, which did not display AHPND symptoms in P. vannamei despite containing the binary toxin genes.

## GENOME ANNOUNCEMENT

Vibrio parahaemolyticus is a halophilic Gram-negative bacterium that has been associated with acute hepatopancreatic necrosis disease (AHPND) in cultured shrimp, Penaeus vannamei. Since its emergence in 2009, the disease has caused severe economic losses in shrimp production in several Southeast Asian countries (1) and more recently in the Americas (2). AHPND-causing V. parahaemolyticus strains harbor Photorhabdus insect-related (Pir) toxin-like genes, and these genes (*pirA-* and *pirB*-like) were shown to be the primary virulence factors in these strains (3).

Recently, the V. parahaemolyticus strain R14 was isolated from P. vannamei shrimp in Latin America that tested positive for harboring the *pir* genes. Despite containing the *pirA* and *pirB* genes, the R14 strain did not cause any mortality in P. vannamei shrimp in a laboratory bioassay, whereas AHPND-causing V. parahaemolyticus strain A3 (the reference strain) caused 100% mortality (4).

V. parahaemolyticus strain R14 was cultured overnight at 30°C on tryptic soy broth. The genomic DNA was extracted using the DNeasy blood and tissue kit (Qiagen, Valencia, CA, USA) following the manufacturer’s protocol. Library preparation and long-read sequencing were carried out at Arizona Genomics Institute, University of Arizona (Tucson, AZ, USA). Approximately 10 µg of total genomic DNA was fragmented to 10 to 20 kbp using the g-TUBE apparatus (Covaris, Woburn, MA, USA) following the manufacturer’s recommendations. A PacBio 20-kb sequencing library was constructed using the SMRTbell template prep kit 1.0 following the manufacturer’s instructions. The final library was processed for sequencing by using PacBio MagBeads kit v 2 with the P6/C4 chemistry and following PacBio protocols (Pacific Biosciences, Menlo Park, CA, USA). Sequencing was performed on a PacBio RS II instrument in one single-molecule real-time (SMRT) cell (v 3) for 6 h. The PacBio hierarchical genome assembly process (HGAP) version 3.0 was used for the *de novo* assembly of the sequence reads (5). An average coverage of 163× was obtained. The genome consists of two chromosomes designated Chr 1 (3,477,001 bp) and Chr 2 (1,818,040 bp) and three plasmids designated pVpR14_74Kb (74,457 bp), pVpR14_56Kb (55,421 bp), and pVpR14_20Kb (19,217 bp).

The genome of the strain was annotated using the RAST version 2.0 pipeline (6). The complete genome consists of 5,444,136 bp, with G+C content of 45.2%, and 4,969 coding sequences and 172 RNAs, of which 37 are tRNAs and 13 are 5S RNAs. The two chromosomes of R14 have an average nucleotide identity of 98.35% with chromosomes of V. parahaemolyticus reference strain RMID2210633 (7). Acquired antimicrobial resistance genes were not detected in any of the plasmids. A beta-lactam resistance signature was found on chromosome 2 of R14 based on the ResFinder 3.0 software program (8). The R14 strain carries a type VI secretion system based on RAST annotation (6).

The *pirAB*-containing region in V. parahaemolyticus R14 strain is encoded on the pVpR14_74Kb plasmid. This novel strain will be an additional repository to the AHPND-causing V. parahaemolyticus collection for functional genomics study.

### Accession number(s).

The complete genome sequence of Vibrio parahaemolyticus strain R14 has been deposited in DDBJ/EMBL/GenBank under the accession numbers CP028141, CP028142, CP028143, CP028144, and CP028145.
